# PRMT6 inhibitors promote fracture healing by modulating osteoclast glucose metabolism

**DOI:** 10.3389/fimmu.2025.1637232

**Published:** 2025-09-18

**Authors:** Nan Wu, Renkai Wang, Shensheng Nian, Hao Zhang, Panyu Zhou, Hao Tang

**Affiliations:** ^1^ Department of Orthopedics, Changhai Hospital, Naval Military Medical University, Shanghai, China; ^2^ Hospital of Orthopedics, General Hospital of Southern Theater Command of PLA, Guangzhou, Guangdong, China

**Keywords:** PRMT6, fracture healing, osteoclasts, glycolysis, EPZ020411

## Abstract

**Introduction:**

Impaired fracture healing remains a significant clinical challenge due to the complexity of the healing process. Osteoclasts, derived from monocytes, are pivotal in bone tissue reconstruction, yet no effective clinical tools exist to promote fracture healing by modulating osteoclast activity.

**Methods:**

We investigated the role of Protein Arginine Methyltransferase 6 (PRMT6) in fracture healing, focusing on its regulation of osteoclast glucose metabolism. PRMT6 deficiency and pharmacological inhibition were employed to assess its functional impact.

**Results:**

PRMT6 was found to promote osteoclastogenesis and activate glycolysis. Both genetic deficiency and pharmacological inhibition of PRMT6 suppressed osteoclast formation and glycolytic activity. Further, PRMT6 inhibitors significantly enhanced fracture healing in vivo by inhibiting osteoclastogenesis.

**Discussion:**

Our study identifies PRMT6 as a key regulator of osteoclast metabolism and fracture repair. Targeting PRMT6 offers a novel therapeutic strategy for impaired fracture healing and provides new insights into the mechanisms of bone tissue regeneration.

## 1 Introduction

Impaired fracture healing is a clinical management challenge. Impaired fracture healing is caused by a variety of reasons, such as aging, chronic inflammation, and metabolic abnormalities (1, 2). Osteoclasts, formed by fusion of monocytes, play a key role in fracture healing. Among them, abnormal activation of osteoclasts is an important cause of impaired fracture healing. Glucose metabolism plays a key role in the activation process of osteoclast formation (3). Glycolysis, as one of the important pathways of cellular energy metabolism, provides necessary energy support for osteoclasts (4, 5). During osteoclast differentiation, glycolysis not only participates in intracellular energy supply but also regulates the generation of inflammatory osteoclasts through energy metabolic switching mechanisms. Inflammatory osteoclasts are abnormal cell types during fracture healing, and their overactivation may lead to delayed or failed fracture healing. It has been shown that RANKL activates HIF1-α (hypoxia-inducible factor 1-α) (6–8) and induces the expression of GLUT1 (glucose transporter protein 1) and glycolytic enzymes In addition, mature osteoclasts have a high rate of glycolysis (9, 10), and glycolysis is an important energy-driven pathway in osteoclast bone resorption. However, the mechanism of osteoclast glucose metabolism regulation is still unclear, so there is no effective clinical treatment to improve fracture healing by regulating osteoclast glucose metabolism.

PRMT6 is an enzyme that plays an important role in various biological processes by methylating the arginine residues of proteins, thereby regulating protein function and stability. PRMT6 deficiency leads to a significant reduction in the expression of key glycolysis enzymes such as PKM (pyruvate kinase), PFKM/L (phosphofructokinase), and LDHA (lactate dehydrogenase), thereby hindering the progression of glycolysis (11, 12). This result suggests that PRMT6 regulates osteoclast differentiation and activation by modulating key enzymes in the glycolysis pathway (12, 13). Recent studies have demonstrated that PRMT6 has a regulatory role in glucose metabolism, and it has been shown that PRMT6 plays a key role in the progression of osteoporosis by promoting osteoclast formation and activation through the regulation of glycolysis. However, the role of PRMT6 in fracture healing has not been reported.

Here, our work demonstrates that PRMT6 is involved in the whole process of fracture healing and that PRMT6 plays a key role in fracture healing-induced osteoclast activation. When PRMT6 was deficient or inhibited, the key proteins of osteoclasts, CTSK (tissue protease K), C-FOS (cellular Fos protein), MMP9 (matrix metalloproteinase 9), and the related genes, NFATc1 (nuclear factor of activated T-cells c1), and DC-STAMP (dendritic cell-specific transmembrane protein), were significantly decreased. Further we demonstrate that utilizing PRMT6 inhibitors EPZ020411 can have the ability to promote rapid fracture healing by inhibiting osteoclast formation. Our work develops new means to promote fracture healing and provides new clues for further understanding of the fracture healing process.

## 2 Materials and methods

### 2.1 Mice

PRMT6-/- purebreds as well as littermate control PRMT6+/+ were obtained by breeding. Tailed genomic DNA PCR was used to characterize mice of the expected genotype. Primers and PCR procedures are detailed in **Table 1**. All mice were raised under specific pathogen-free conditions. All animal experiments were performed in accordance with the National Institutes of “Health Guide for the Care and Use of Laboratory Animals “and approved by the Medical Ethics Committee of the Naval Medical University.

**Table 1:** Primers list.

Genes	Forward (5’-3’)	Reverse (3’-5’)
GAPDH	ACTGAGGACCAGGTTGTC	TGCTGTAGCCGTATTCATTG
CTSK	GGAGTTGACTTCCGCAATCCT	ACTTGAACACCCACATCCTGC
DC-stamp	GGAGAGTCCGAGAATCGAGAT	TTGCAGCTAGGAAGTACGTCT
PRMT6	GATGGGCTACGGACTTCTGC	GCATCTGGTCGCTAATCGGG

### 2.2 Osteoclast differentiation *in vitro*

Bone marrow-derived monocytes/macrophages (BMMs) were obtained from the femurs and tibias of mice. The bone marrow was flushed out and cultured in α-MEM medium (Gibco, USA) supplemented with 25 ng/ml Macrophage Colony-Stimulating Factor (M-CSF, Peprotech, USA) and 50 ng/ml Receptor Activator of Nuclear Factor-κB Ligand (RANKL, Peprotech, USA) for 7 days. The cells were incubated in a humidified incubator containing 5% CO^2^ at 37°C, with medium changes every two days. BMMs were treated with different concentrations of PRMT6 inhibitor (EPZ020411, MCE, USA), and when osteoclasts were fully differentiated, they were stained using a TRAP (Tartrate-Resistant Acid Phosphatase) staining kit (Servicebio) (14).

### 2.3 Micro-CT

To investigate the impact of PRMT6 deficiency on fracture healing in mice, Micro-CT was employed to examine the microstructural changes in the femurs of fractured mice. In brief, femurs from five mice per group were collected, dissected to remove excess soft tissue, and then fixed in 4% paraformaldehyde for 48 hours. The instrument settings were as follows: resolution, 10 µm/pixel; voltage, 40 kV; current, 250 µA; exposure time, 0.9 seconds; rotation step, 0.4˚, with a total rotation of 180˚. Within the callus region, bone parameters were measured, including bone volume fraction (BV/TV), trabecular number (Tb.N), trabecular thickness (Tb.Th), trabecular separation (Tb.Sp), and bone mineral density (BMD).

### 2.4 Western blotting

To extract total protein, BMMs were lysed in RIPA lysis buffer (catalog number PC103, Yamei Biotechnology Co., Ltd.) containing Protease and Phosphatase Inhibitor Cocktail (100X, catalog number GFR103, Yamei Biotechnology Co., Ltd.). The concentration of total protein was quantified using a BCA Protein Quantitation Kit (catalog number ZJ101, Yamei Biotechnology Co., Ltd.). Equal amounts of total protein were loaded into each lane for sodium dodecyl sulfate-polyacrylamide gel electrophoresis (SDS-PAGE) and then transferred onto polyvinylidene difluoride (PVDF) membranes. The membranes were blocked in 1xTBST solution (Servicebio Technology Co., Ltd.) for 1 hour and subsequently incubated overnight at 4°C with primary antibodies specific to PRMT6, CTSK (Cathepsin K), C-FOS (Cellular FOS Protein), MMP9 (Matrix Metalloproteinase-9), or GAPDH. After washing with 1xTBST, the membranes were incubated with secondary antibodies at room temperature for 1 hour. Signals were detected using a Western Blot Imaging System (ibright FL1500, Invitrogen, Singapore), and band intensities were normalized to GAPDH and quantified using ImageJ.

### 2.5 qRT-PCR

According to the manufacturer’s instructions (15), total RNA was extracted using the RNeasy Kit (Vazyme; Catalog No. 75142). cDNA was synthesized from 1 microgram of RNA using the Omniscript Reverse Transcriptase Kit (Vazyme; Catalog No. 205113). qPCR was performed on a QuantStudio 3 (Applied Biosystems, Applied Biosystems, Warrington, UK; ThermoFisher Scientific), and the relative mRNA concentrations were calculated using the delta-delta comparative threshold cycle (2-ΔΔCt) method, normalized to those of NFATc1, DC-STAMP, and PRMT6, which can be seen in **Table 2**.

**Table 2:** Workflow of PCR.

Stage 1	Premutability^a^	Rep:1	95°C	30sec
Stage 2	cyclic response	Reps: 40	95°C	3–10 sec^b^
			60°C	10–30 sec^c^
			95°C	15 sec
Stage 3	melting^d^	Rep: 1	60°C	60 sec
			95°C	15 sec

### 2.6 CCK8

According to the manufacturer’s instructions, the CCK-8 Kit (Catalog No. C0038; Dojindo Laboratories, Japan) was used to determine the effects of EPZ020411 on the proliferation and viability of bone marrow-derived macrophages (BMMs). Cells were seeded in 96-well plates at a density of 1x10^5^ cells per well and incubated overnight. Subsequently, α-MEM medium containing various concentrations (0, 5, and 10 µM) of EPZ020411 was added, and the cells were incubated for 24 hours at 37°C. Afterward, 10 µl of CCK-8 buffer was added to each well, and the cells were further incubated for 2 hours at 37°C. The optical density (OD) was then measured at 450 nm using a Multiskan absorbance microplate reader (Thermo Fisher Scientific, Inc.). Cell viability was calculated using the following formula: Cell viability = (Experimental group OD - Blank OD)/(Control group OD - Blank OD).

### 2.7 Femur fracture model

Firstly, six-week-old PRMT6^+/+^ mice (control group) and PRMT6^-/-^ mice (experimental group) were anesthetized according to the guidelines of the Animal Ethics Committee of Navy Medical University of Chinese People’s Liberation Army (15, 16). The hair on the surface of the skin on the right lower limb was removed, followed by disinfection of the local skin. An incision was then made along the femur, and a syringe needle was inserted into the bone marrow space through the incision made along the femur. Osteotomy was performed after transecting the femur, and the incision was closed using 5–0 silk sutures.

### 2.8 RNA-seq

RNA-Seq process begins with the extraction of total RNA using Trizol reagent. Subsequently, mRNA is enriched through Oligo(dT) beads and fragmented into short segments. These mRNA fragments are then reverse transcribed into cDNA using random primers. Purification of the cDNA fragments is achieved using the QiaQuick PCR extraction kit, followed by end repair, poly(A) tail addition, and ligation to Illumina sequencing adapters. The ligation products undergo size selection via agarose gel electrophoresis, PCR amplification, and are sequenced using the Illumina HiSeq™ 2500 platform. To ensure data quality, raw reads are filtered to exclude those containing adapters, more than 10% unknown nucleotides, or more than 50% low-quality bases (Q-value ≤ 20). Subsequently, rRNA-mapped reads are removed using the Bowtie2 short read alignment tool. The remaining reads are mapped to the reference genome using TopHat2 (version 2.0.3.12). Gene abundance is quantified using RSEM software and normalized by the FPKM (Fragments Per Kilobase of transcript per Million mapped reads) method.

### 2.9 Statistical Analysis

All data were analyzed using SPSS 24.0 (IBM, Armonk, USA). Statistic difference was analyzed by unpaired two-tailed Student’s t test or by a one-way analysis of variance (ANOVA) followed by the lest significant difference test. A p-value < 0.05 was considered statistically significant.

## 3 Results

### 3.1 PRMT6 is involved in the process of fracture healing

**Figure 1:**
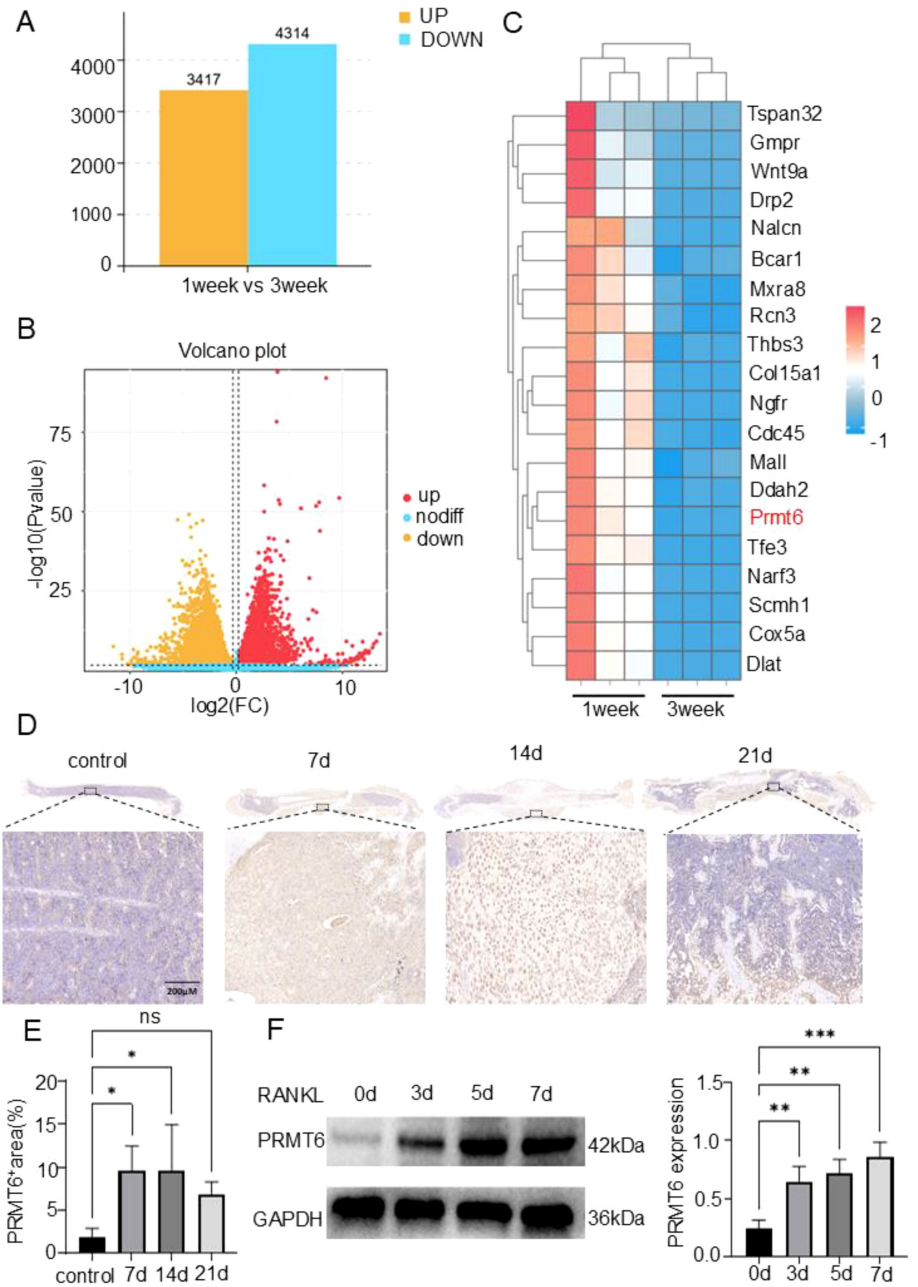
PRMT6 is a key regulatory gene that can participate in the process of bone fracture healing. **(A, B)** Column chart and volcano plot of differentially expressed genes (DEGs) in bone callus at 1 week and 3 weeks after fracture. **(C)** Heatmap of gene expression values at the intersection of 1 week and 3 weeks after fracture for 20 genes. **(D, E)** Immunohistochemistry to verify the expression of PRMT6 in bone tissue of C57 mice as the fracture time increases. **(F)** Western blotting to detect changes in the expression of PRMT6 during osteoclast differentiation. (n=3, p ≤ 0.05). *p < 0.05, **p < 0.01, ***p < 0.001; "ns" is P<0.05.

In search of crucial regulatory genes in the process of bone fracture healing, C57 mice were modeled for fracture and analyzed by RNA sequencing one week versus three weeks after fracture. The analysis showed a total of 7731 differentially expressed genes (DEGs) (FC>1.5, P<0.05), of which, 4314 were down-regulated and 3417 were up-regulated (**Figures 1A, B**). Subsequently, heatmap analysis showed that PRMT6 expression was significantly reduced in bone tissues of C57 mice three weeks after fracture compared with one week after fracture (**Figure 1C**). Meanwhile, immuno-histochemical staining corroborated the previous observation that PRMT6-positive areas were barely visible in the bone tissues of unfractured mice, whereas the expression of PRMT6 increased gradually in a time-dependent manner in C57 mice at 7 and 14 days after fracture9 (**Figures 1D, E**). However, it is noteworthy that at 21 days of fracture, there was a clear trend of decreasing PRMT6 expression (**Figures 1D, E**). At the cellular level, we extracted mononuclear macrophages (BMMs) from C57 mice and exposed them to RANKL, and the protein level of PRMT6 was again verified by WB experiments at induction days 0, 3, 5, and 7, and was progressively up-regulated over time in RANKL-induced BMMs (**Figure 1F**).

Overall, these results established that PRMT6 is able to participate in the fracture healing process, and that its expression changes with time and is a key regulatory gene in the fracture healing process.

### 3.2 PRMT6 gene knockout mice can promote fracture healing

**Figure 2:**
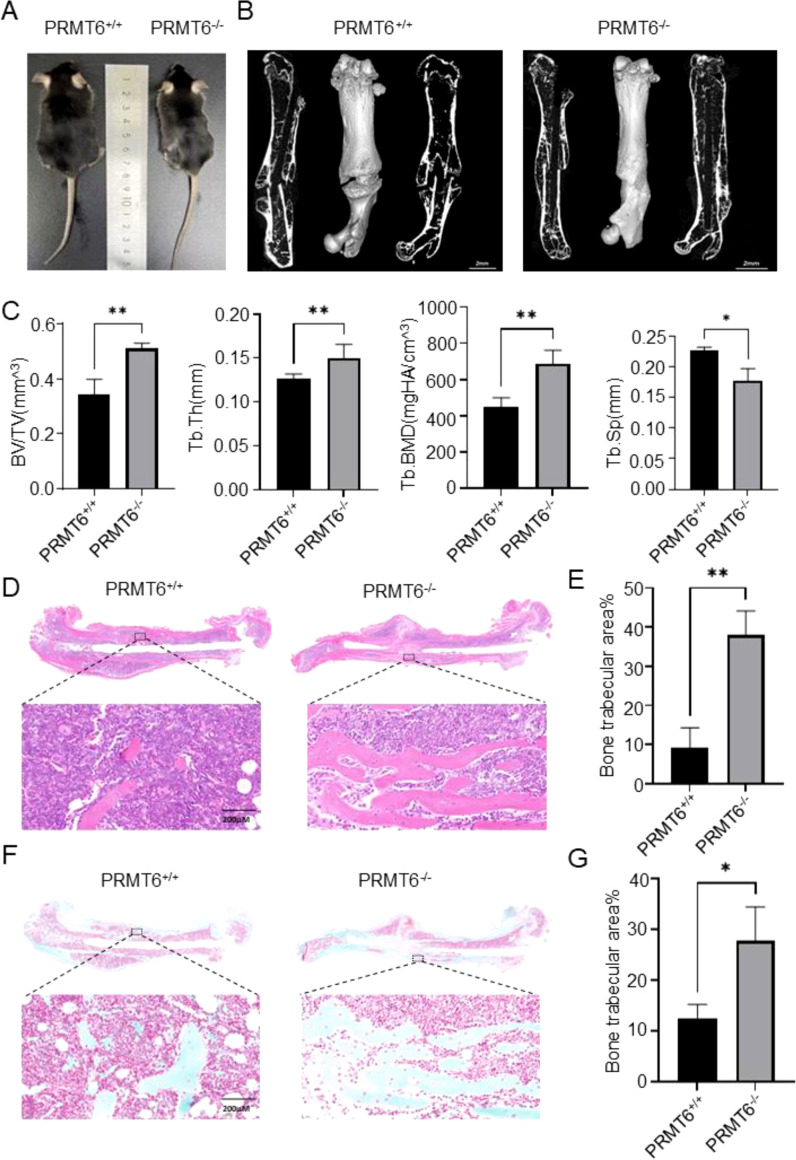
PRMT6 gene deficiency mice can promote fracture healing. **(A)** Representative photograph of PRMT6^-/-^ mice and PRMT6^+/+^ control littermate at 8 week of age. **(B)** Knockout mice group and control group fracture sites were reconstructed using micro-CT on day 21. **(C)** Quantitative analysis of BV/TV, Tb.Th, Tb.BMD and Tb.Sp levels. **(D)** Representative images of HE staining. **(E)** Quantitative analysis of the bone trabecular area in the fracture site area. **(F)** Representative images of Sarfranin O-Fast Green staining. **(G)** Quantitative analysis of the mature bone trabecular area in the fracture site area. (male, n=5, p ≤ 0.05). *p < 0.05, **p < 0.01.

Given that PRMT6 can participate in the fracture healing process, we investigated the role of PRMT6 in the fracture healing process. We first compared PRMT6+/+ mice with PRMT6-/- mice, and it could be observed that PRMT6-/- mice were slightly smaller than PRMT6+/+ mice in appearance (**Figure 2A**). The mice were subsequently genotyped, and the mouse genotypes were analyzed based on the identification results (**Supplementary Figure S1A**) and verified by Western Blot (**Supplementary Figure S1B**). The mice screened based on genotype identification were then subjected to Micro-CT experiments, which showed that three weeks after fracture, PRMT6-/- mice exhibited elevated bone volume fraction (BV/TV), trabecular bone thickness (Tb.Th), and trabecular bone mineral density (Tb.BMD) when compared with the control PRMT6+/+ group, and it is noteworthy that trabecular bone separation (Tb. Sp) did decrease slightly (**Figures 2B, C**), which, based on previous findings, can fully demonstrate that knockdown of the PRMT6 gene can accelerate the fracture healing process in mice.

These findings were further confirmed by histomorphometric staining analysis. In HE staining, we observed a significant increase in bone trabecular area in PRMT6-/- mice compared with PRMT6+/+ mice (**Figures 2D, E**). And again in SO-FG staining, confirming the previous observation, the area of bone trabeculae developed from chondrocytes was significantly increased in PRMT6-/- mice compared with mice in the PRMT6+/+ group (**Figures 2F, G**). Notably, in this process, the number of osteoclasts was significantly reduced after PRMT6 knockdown compared with PRMT6 no knockdown, suggesting that PRMT6 promotes osteoclast differentiation and activation (**Supplementary Figure S1C**).

Taken together, these results demonstrate that PRMT6 can promote osteoclast differentiation *in vivo* and promote fracture healing in the presence of PRMT6 knockdown.

### 3.3 Glycolysis is a key signaling pathway that influences the process of fracture healing

**Figure 3:**
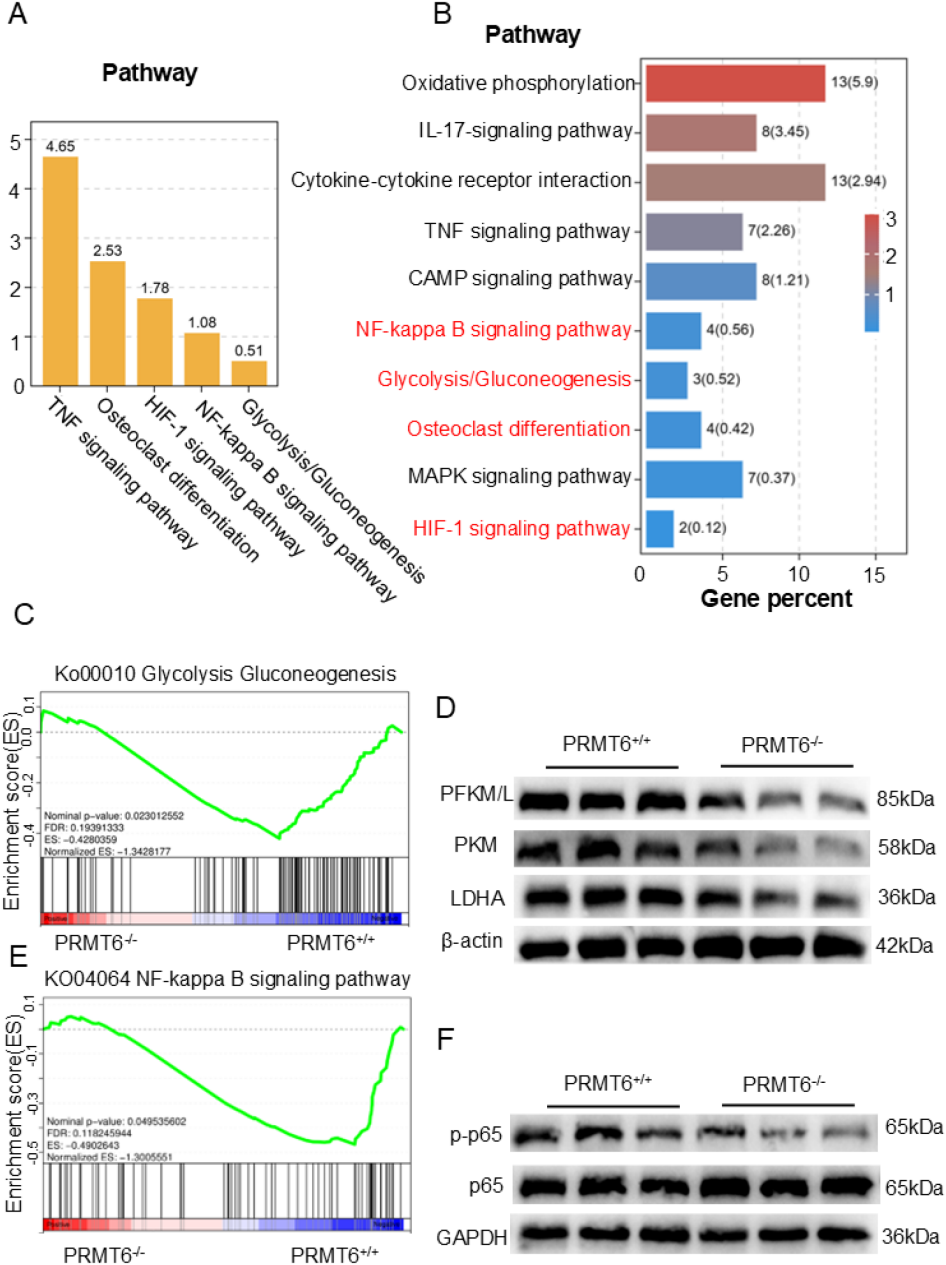
Glycolysis can play a crucial role in the process of fracture healing. **(A)** RNA-seq data analysis revealed the expression of KEGG signaling categories and the glycolysis pathway in Prmt6−/− fracture mice compared to Prmt6+/+ mice. **(B)** Based on the differentially expressed genes (DEGs) between Prmt6−/− and Prmt6+/+ fracture mice, the top 10 significantly enriched KEGG pathways were identified from the RNA-seq data. **(C)** GSEA analysis showed that, compared to Prmt6+/+ mice, Prmt6−/− mice exhibited significant inhibition of the glycolysis pathway three weeks after fracture. **(D)** WB analysis confirmed that the expression of glycolysis-related enzymes (PFKM/L, PKM, and LDHA) was inhibited in Prmt6−/− bone marrow-derived macrophages (BMMs) compared to PRMT6+/+ BMMs. **(E)** GSEA analysis indicated that, compared to PRMT6+/+ mice, Prmt6−/− mice exhibited significant inhibition of the NF-κB pathway three weeks after fracture. **(F)** WB analysis revealed that the expression of p-p65 was inhibited in Prmt6−/− bone marrow-derived macrophages (BMMs) compared to PRMT6+/+ BMMs.

To further investigate the specific molecular mechanisms by which PRMT6 affects fracture healing, PRMT6-/- mice PRMT6+/+ mice were subjected to fracture modeling, and RNA-seq analysis was performed three weeks after fracture. Principal component analysis (PCA) showed that samples before and after PRMT6 knockdown clustered significantly, indicating significant transcriptional changes (**Supplementary Figure S1D**). The analysis identified 461 differentially expressed genes (DEGs) (FC ≥ 1.5, *P* < 0.05), of which 253 were up-regulated and 208 were down-regulated (**Supplementary Figure S1E**). Subsequent gene ontology (GO) enrichment analysis of these DEGs highlighted significant enrichment in a variety of biological processes, with a focus on GO terms associated with metabolic regulation and molecular mechanisms (**Supplementary Figure S2A**). In addition, in the KEGG significance bar graph, the signaling pathway of osteoblast differentiation had a strong influence, while signaling pathways such as glycolysis/glycolysis, NF-κB, and HIF-1 were also significantly enriched (**Figure 3A**). We observed the same results in the KEGG enrichment bar graph, where RNA-seq analysis of DEGs performed three weeks after fracture in mice showed significant enrichment of these pathways among the first 10 identified pathways (**Figure 3B**). Notably, differential peak analysis between Prmt6-/- and Prmt6+/+ fractured tissues showed significant enrichment of the HIF-1 signaling pathway that mediates glycolysis and the osteoclast differentiation KEGG pathway, with the highest enrichment factor (**Supplementary Figure S2B**).

Considering the role of HIF-1 in being able to mediate the onset of glycolysis and the enrichment observed in the glycolysis/glycolysis pathway, we explored how PRMT6 deficiency affects glycolysis during fracture healing. Gene set enrichment analysis (GSEA) of RNA-seq data three weeks after fracture showed a significant reduction in glycolysis in the PRMT6-/- group (**Figure 3C**). Meanwhile, Western Blot analysis supported this observation by showing that key glycolytic enzymes (PFKM/L, PKM, and LDHA) were significantly inhibited in PRMT6-/- cells after RANKL stimulation (**Figure 3D**, **Supplementary Figures S2C–E**).

Based on previous studies we learned that NF-κB is a key signaling pathway for osteoblast differentiation, so we explored its activation in the absence of PRMT6, and GSEA showed that the NF-κB signaling pathway was significantly activated in PRMT6+/+ samples (**Figure 3E**). We observed the same results in Western Blot, which led to rapid phosphorylation of p65 upon RANKL stimulation in PRMT6+/+ cells (**Figure 3F**, **Supplementary Figure S2F**).

Collectively, these results suggest that PRMT6 promotes osteoclastogenesis and that glycolysis is an important signaling pathway in this process.

### 3.4 PRMT6 deficiency inhibits differentiation and activation in osteoclasts in mouse bone marrow macrophages

**Figure 4:**
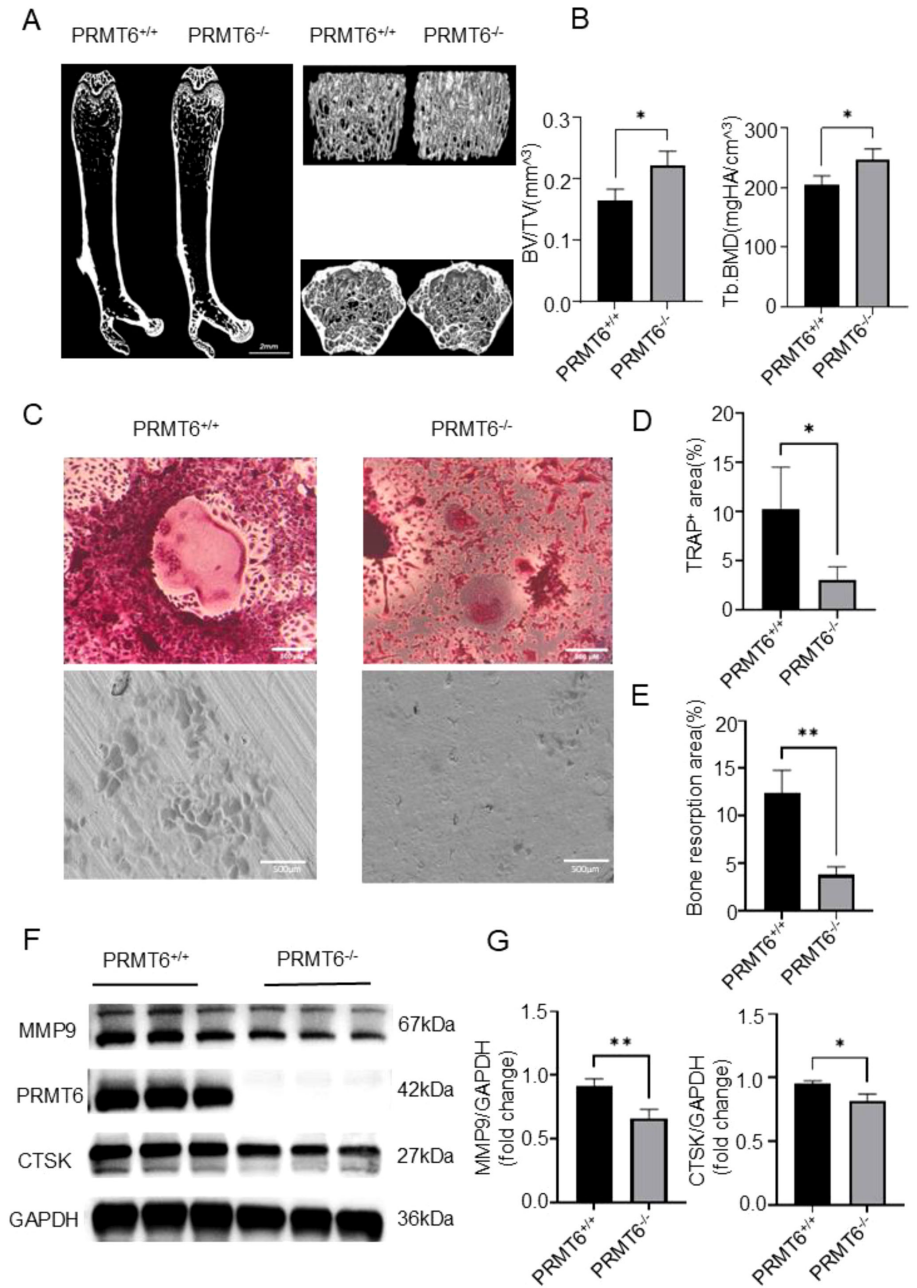
PRMT6 deficiency can suppress osteoclast differentiation and activation in mice BMMs. **(A)** Representative computer renderings of bone structure in the femurs from PRMT6^-/-^ and PRMT6^+/+^mice at 8 week. (male, n = 3). **(B)** Quantitative analysis of BV/TV, and Tb.BMD levels. **(C-E)** BMMs were from of PRMT6^-/-^ mice and PRMT6^+/+^ control littermate. TRAP staining and a bone resorption assay were conducted to assess the osteoclast differentiation, quantitative analysis of the TRAP^+^ area and bone resorption area. **(F, G)** Western blotting was conducted to detect the expression of MMP9, PRMT6 and CTSK. (scale bar =500μm, p ≤ 0.05). *p < 0.05, **p < 0.01.

To investigate the role of PRMT6 in osteoclast differentiation induced by BMMs, we first subjected PRMT6+/+ mice without any intervention to Micro-CT scanning compared with PRMT6-/- mice, which showed that PRMT6-/- mice had significantly elevated BV/TV, Tb.BMD compared with PRMT6+/+ mice (**Figures 4A, B**). Induction was performed according to a brief flow chart of osteoclast induction (**Supplementary Figure S3A**). Immediately afterward, we observed that large-area and well-developed osteoclasts could be formed in BMMs of PRMT6+/+ mice with significant bone resorption (**Figures 4C–E**). However, osteoclasts were significantly inhibited in BMMs of PRMT6-/- mice, as evidenced by the small area of mature osteoclasts and significantly reduced bone resorption activity (**Figures 4C–E**). In addition, we demonstrated that in the absence of PRMT6 (**Supplementary Figure S3B**), the levels of osteoclast-associated proteins CTSK and MMP9 were reduced and vice versa (**Figures 4F, G**).

Collectively, these results suggest that PRMT6 promotes RANKL-induced osteoclastogenesis *in vitro* and inhibits the generation of bone trabeculae, a key component of fracture healing, *in vivo*.

### 3.5 PRMT6 inhibitor EPZ020411 inhibits differentiation and activation in osteoclasts in mouse bone marrow-derived macrophages

**Figure 5:**
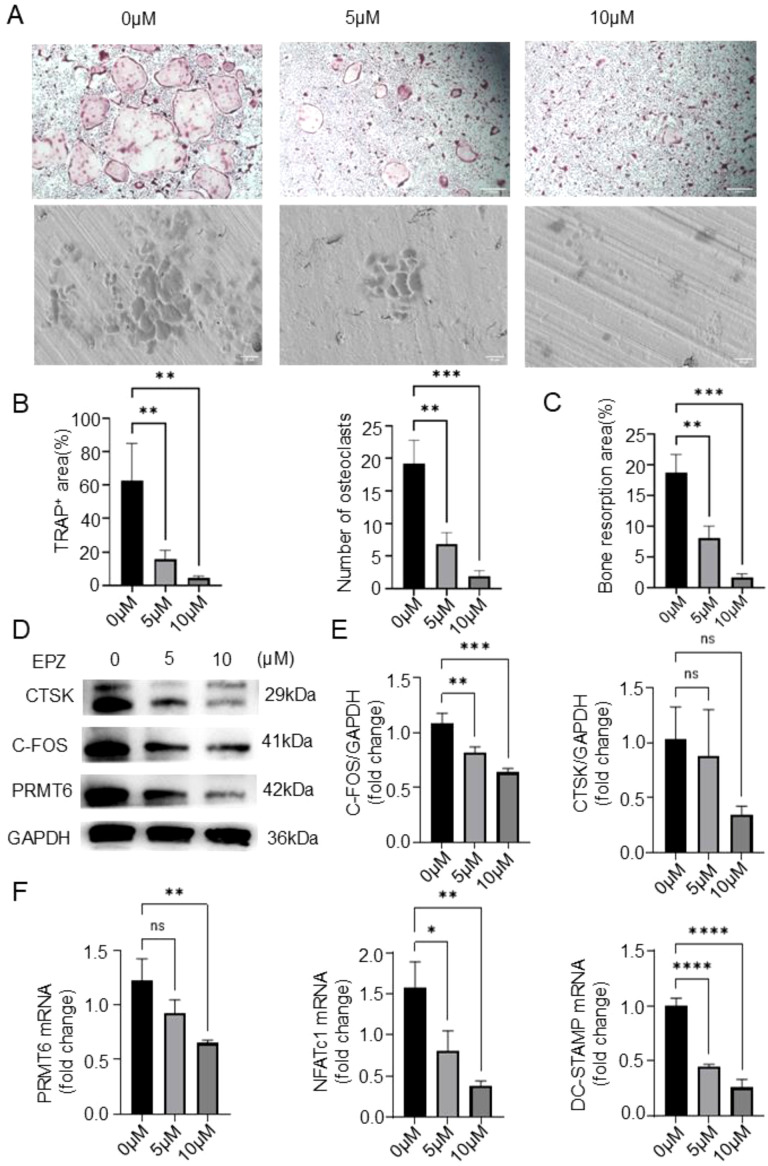
PRMT6 inhibitor EPZ020411 can suppress osteoclast differentiation and activation in mice BMMs. **(A-C)** BMMs were treated with EPZ020411 of different concentrations therapy. TRAP staining and a bone resorption assay were conducted to assess the osteoclast differentiation, and quantitative analysis of the TRAP^+^ area,number of osteoclasts and bone resorption area. **(D, E)** Western blotting was conducted to detect the expression of C-FOS,PRMT6 and CTSK. **(F)** Q-PCR analysis of gene expression levels of osteoclast markers (NFATc1 and DC-STAMP) in RANKL-induced BMMs after PRMT6 inhibition.(scale bar =200μm, p ≤ 0.05). *p < 0.05, **p < 0.01, *** p < 0.001, **** p < 0.0001; "ns" is P<0.05.

Previously, we demonstrated the ability to inhibit osteoclastogenesis in the absence of PRMT6, and to further investigate the role of PRMT6 in RANKL- induced osteoclastogenesis, we used a selective PRMT6 inhibitor, EPZ020411, in our experiments. Subsequently, in order to determine whether the inhibitory effect of PRMT6 deficiency on osteoclast formation was associated with cytotoxicity, the BMMs viability assays were performed using Cell Counting Kit-8 assays (CCK-8). The results obtained indicated that EPZ020411 did not produce cytotoxicity on BMMs at concentrations lower than 15 µM (**Supplementary Figure S3C**), so we finally chose drug concentrations of 0, 5, and 10 µM for subsequent experiments. Notably, PRMT6 inhibition resulted in a dose-dependent reduction in osteoclast formation, as evidenced by a decrease in the number and area of osteoclasts (**Figures 5A, B**), as well as a dose-dependent significant reduction in bone resorption activity (**Figures 5A–C**). Furthermore, in the context of PRMT6 inhibition (**Supplementary Figure S3D**), it also led to a reduction in the levels of osteoclast-associated proteins CTSK and C-FOS (**Figures 5D, E**) and, consequently, osteoclast-associated genes, DC-STAMP and NFATC1 (**Figure 5F**).

Collectively, these results suggest that PRMT6 promotes RANKL-induced osteoclastogenesis *in vitro*.

### 3.6 PRMT6 inhibitor EPZ020411 accelerates the fracture healing process

**Figure 6:**
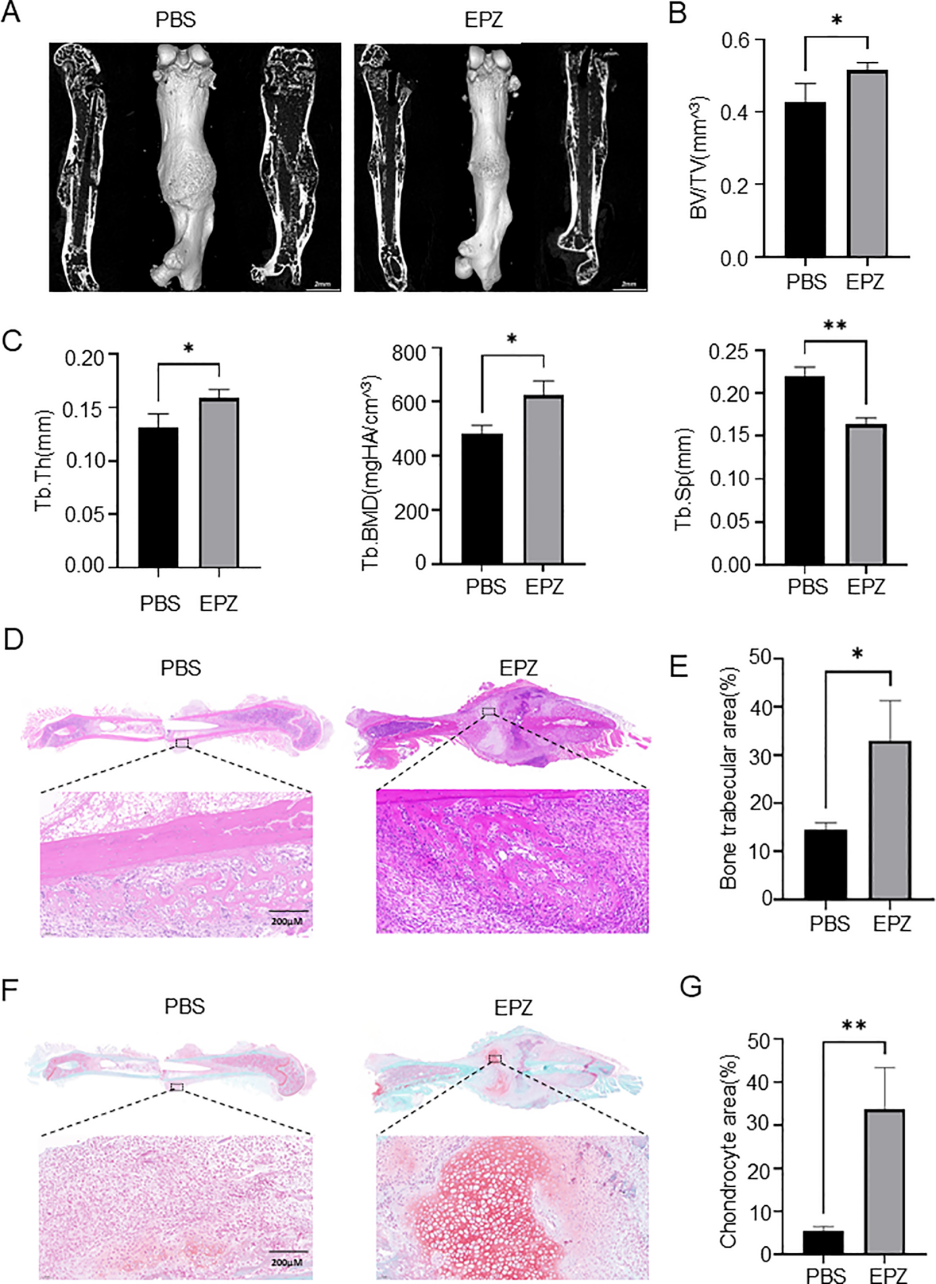
PRMT6 inhibitor EPZ020411 can accelerate the process of fracture healing **(A)** Using micro-CT reconstruction images of the fracture site at 21 days after fracture in mice injected with PBS and mice injected with the inhibitor EPZ020411. (male, n = 3). **(B, C)** Quantitative analysis of BV/TV, Tb.Th, Tb.BMD and Tb.Sp levels. **(D, E)** Representative HE staining images of the inhibitor group and the PBS group, quantitative analysis of the bone trabecular area. **(F, G)** Representative Sarfranin O-Fast Green staining images of the inhibitor group and the PBS group, quantitative analysis of the chondrocyte area.

Considering our previously mentioned critical role of PRMT6 in promoting osteoclastogenesis *in vivo* and *in vitro*, we investigated it as a potential target for fracture healing therapy. We fabricated a previously described consistent fracture model using the selective PRMT6 inhibitor EPZ020411 and administered it for 7 consecutive days, and for the control group we chose to inject PBS. a brief flowchart is shown in **Supplementary Figure S3E**. Subsequent Micro-CT assays were performed, and the results showed that three weeks after fracture, mice injected with the inhibitor exhibited elevated bone volume fraction (BV/TV), trabecular thickness (Tb.Th), and trabecular density (Tb.BMD), and notably, trabecular segregation (Tb.Sp) was significantly reduced compared with the PBS-injected group (**Figures 6A–C**). These data suggest that the PRMT6 inhibitor EPZ020411 was able to accelerate fracture healing.

In addition, histological staining was performed one week after the injection of the drug, and we further confirmed the previous results, and in HE staining, a significant increase in trabecular area could be observed in fracture tissues injected with EPZ020411 compared to bone tissues injected with PBS (**Figures 6D, E**). Moreover, in SO-FG staining, we found that chondrocyte area was significantly increased in fracture tissues when PRMT6 was inhibited compared to when it was not inhibited (**Figures 6F, G**). Interestingly, from TRAP staining we found that the number of osteoclasts was instead increased in the early stage of fracture in the presence of PRMT6 inhibition. (**Supplementary Figure S3F**). This phenomenon may be associated with the release of inflammatory factors following fracture, which include TNF-α, IL-1β, and IL-6, among others. These cytokines are not only involved in the regulation of inflammatory responses but also impact the formation and activity of osteoclasts. For example, TNF-α can promote the differentiation and maturation of osteoclasts via the RANKL pathway, while IL-6 exerts pro-inflammatory effects in the early stages of fracture healing and can enhance the formation of osteoclasts.

Taken together, these results demonstrate that inhibition of PRMT6 effectively reduces osteoclastogenesis and trabecular bone loss during fracture healing. This highlights PRMT6 as a promising therapeutic target for fracture healing treatment as well as for bone nonunion.

## 4 Discussion

Fracture is the most common and most frequent disease in orthopedics, and 5-10% of fracture patients worldwide suffer from non-union, delayed healing, and nonunion due to various factors. Therefore, impaired healing after fracture is an important clinical problem in trauma orthopedics, and the study of its mechanism is of great significance to the clinical fracture treatment strategy. Fracture healing is a complex system that is regulated by multiple signaling pathways (17, 18), in which the synergistic effect between osteoclasts and osteoblasts plays a central role. Osteoblasts regulate the recruitment, adhesion, proliferation and differentiation of osteoclasts, while osteoclasts regulate the activity and function of osteoblasts. However, a growing body of research suggests that aberrant activation of osteoclasts not only exacerbates the inflammatory response, but also impedes fracture healing Osteoclast formation is regulated by several pathways, the well-known one being the NF-κB pathway. However, recent studies have shown that glucose metabolism is becoming an important pathway affecting osteoclastogenesis. Because most of the energy required for osteoclastogenesis is provided by glycolysis, which is the main way for cells to obtain energy under hypoxic or low oxygen conditions.

PRMT6 is an enzyme involved in post-translational modification of proteins, which regulates the function and stability of proteins mainly through methylation modification of arginine residues. Recent studies have shown that PRMT6 plays an important role in a variety of biological processes, including cell proliferation, differentiation, apoptosis, and immune responses (19, 20). However, little has been reported about the role of PRMT6 in fracture healing. In the present study, transcriptome sequencing analysis revealed that the expression of PRMT6 was significantly down-regulated during fracture healing, suggesting that it may be involved in this process. Further studies showed that PRMT6 could promote osteoclastogenesis and activation, thereby accelerating the process of bone resorption at the fracture site, which in turn affected subsequent bone regeneration. In addition, this study found that PRMT6 was able to activate the glycolytic process and promote osteoclast differentiation. Specifically, up-regulation of PRMT6 expression was able to increase the expression and activity of key enzymes of glycolysis, thereby accelerating glucose catabolism and energy production (21, 22). This process provides the necessary energy support for osteoclast differentiation and activation. Among these, the mechanism by which PRMT6 regulates key glycolytic enzymes may be closely related to the HIF-1 signaling pathway. The HIF-1 signaling pathway is a key pathway mediating glycolysis and is capable of upregulating key glycolytic enzymes such as pyruvate kinase. Research has found that the expression level of the HIF-1 signaling pathway is suppressed in PRMT6−/− BMMs, which indicates that PRMT6 affects glycolysis levels in osteoclasts by regulating the HIF-1 signaling pathway. In addition, PRMT6 influences gene transcription activity by methylating histone H3R2me2a. This epigenetic regulation can also affect the expression of glycolysis-related genes. However, it is worth noting that glycolysis may have a dual role in osteoclast differentiation. On the one hand, glycolysis provides the necessary energy support for osteoclast differentiation; on the other hand, metabolites and signaling molecules during glycolysis may also be involved in regulating osteoclast differentiation and function. However, excessive glycolysis may also lead to the generation of inflammatory osteoclasts, which is detrimental to fracture healing. This finding reveals the important role of PRMT6 in regulating osteoclast differentiation and function, and provides a theoretical basis for its application in fracture healing.

To further investigate the role of PRMT6 in fracture healing, this study constructed a mouse model with PRMT6 deficiency or inhibition and observed its effect on fracture healing. The results showed that PRMT6 deficiency or inhibition significantly inhibited RANKL-induced osteoclastogenesis and glycolysis processes, thereby slowing down the bone resorption process at the fracture site. This effect favored the reduction of inflammatory response and excessive bone resorption at the fracture site, providing a better environment for subsequent bone regeneration and repair. As a result, PRMT6-deficient or inhibited mice exhibited faster healing and better healing quality during fracture healing. Therefore, PRMT6 is expected to be a new target for fracture healing and bone nonunion treatment and a candidate gene for new drug development.

## 5 Conclusion

In this study, we revealed the critical role of PRMT6 in RANKL-induced osteoclastogenesis and fracture healing by transcriptional sequencing analysis and other methods, and explored the mechanism by which it affects osteoclastogenesis and function by regulating glycolysis. This finding provides new ideas and targets for the treatment of fracture healing and bone nonunion. In the future, we will continue to delve into the specific mechanism of PRMT6’s role in fracture healing and explore the prospects for drug development and clinical application targeting PRMT6. Meanwhile, we will also focus on other metabolic pathways and signaling pathways that may affect fracture healing, with the aim of providing a more comprehensive and in-depth understanding of the treatment of fracture healing and related diseases.

## Data Availability

The original contributions presented in the study are publicly available. This data can be found here: https://www.ncbi.nlm.nih.gov/sra/PRJNA1321902, accession number PRJNA1321902.
