# Supplementary figures and images for: PRMT6 inhibitors promote fracture healing by modulating osteoclast glucose metabolism
Source: Front Immunol. 2025 Sep 18;16:1637232. doi: 10.3389/fimmu.2025.1637232 (PMC12488595; doi:10.3389/fimmu.2025.1637232)

**Figure 1 E**

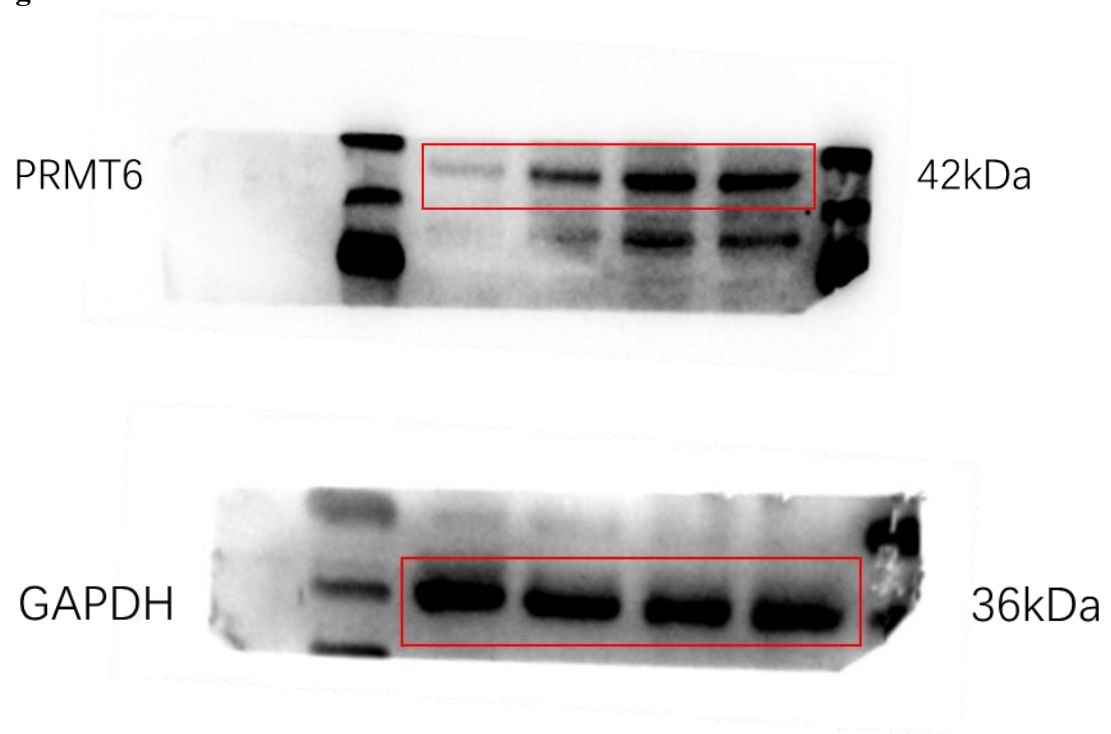

**Figure 3D**

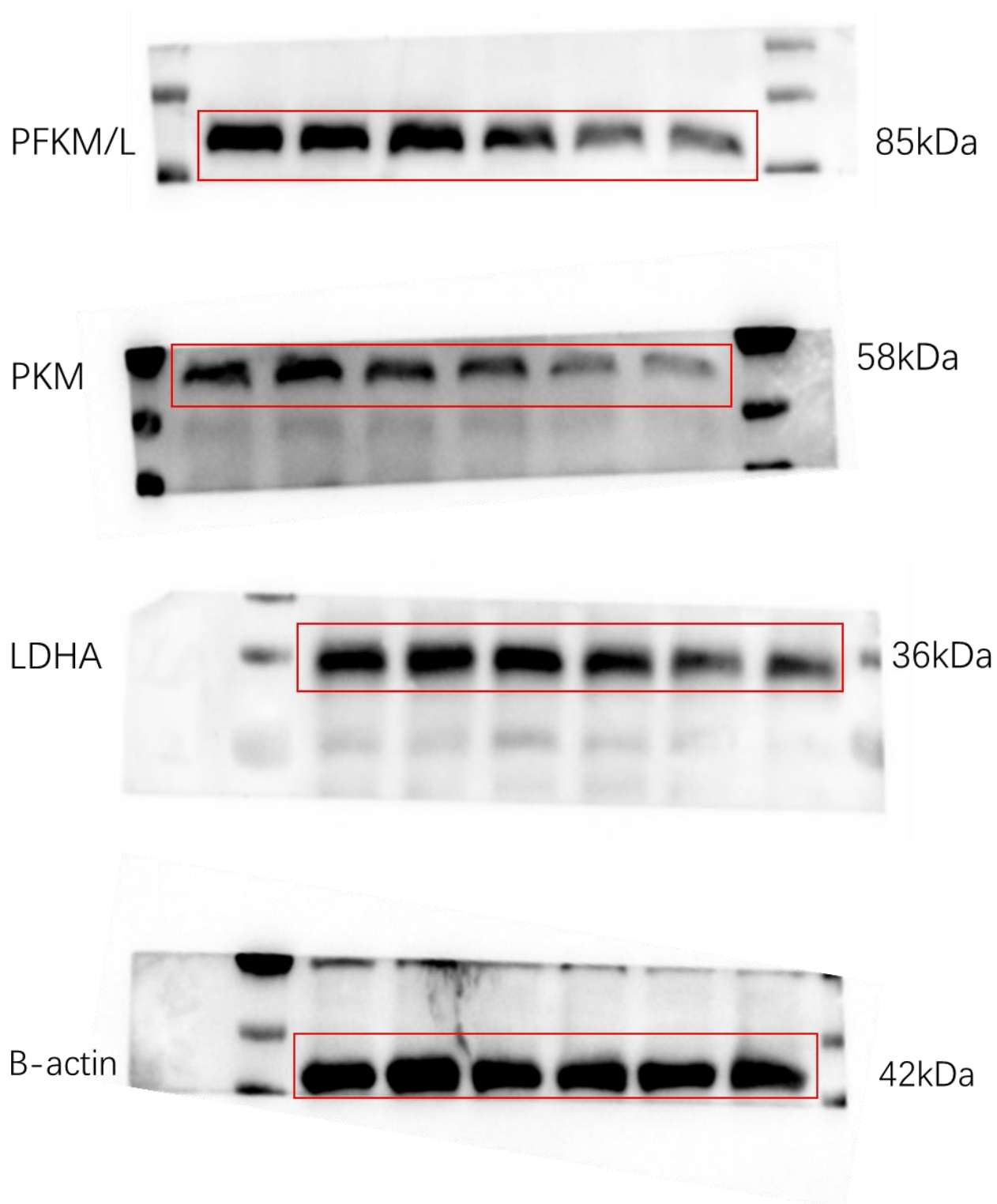

**Figure 3F**

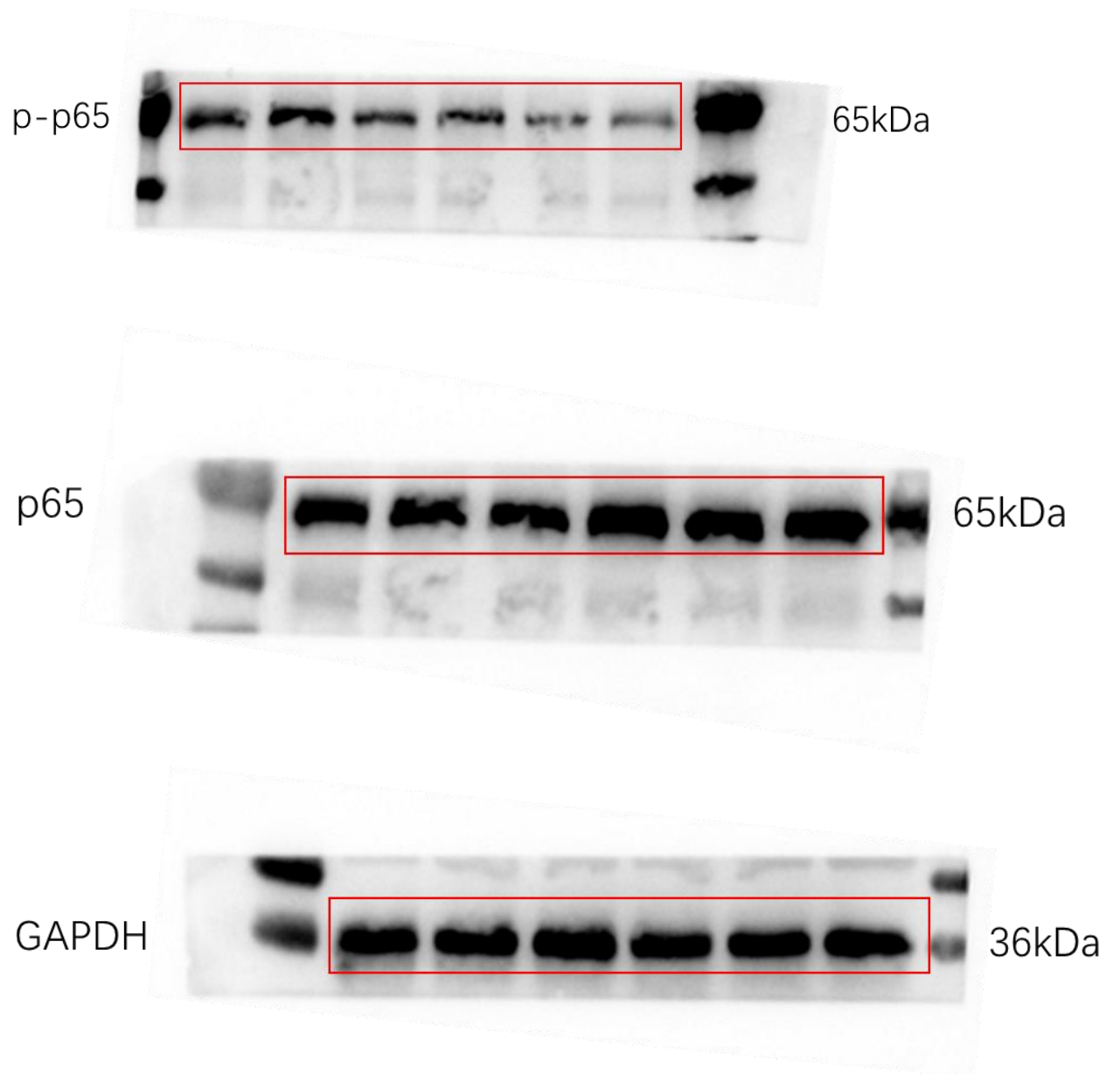

**Figure 4F**

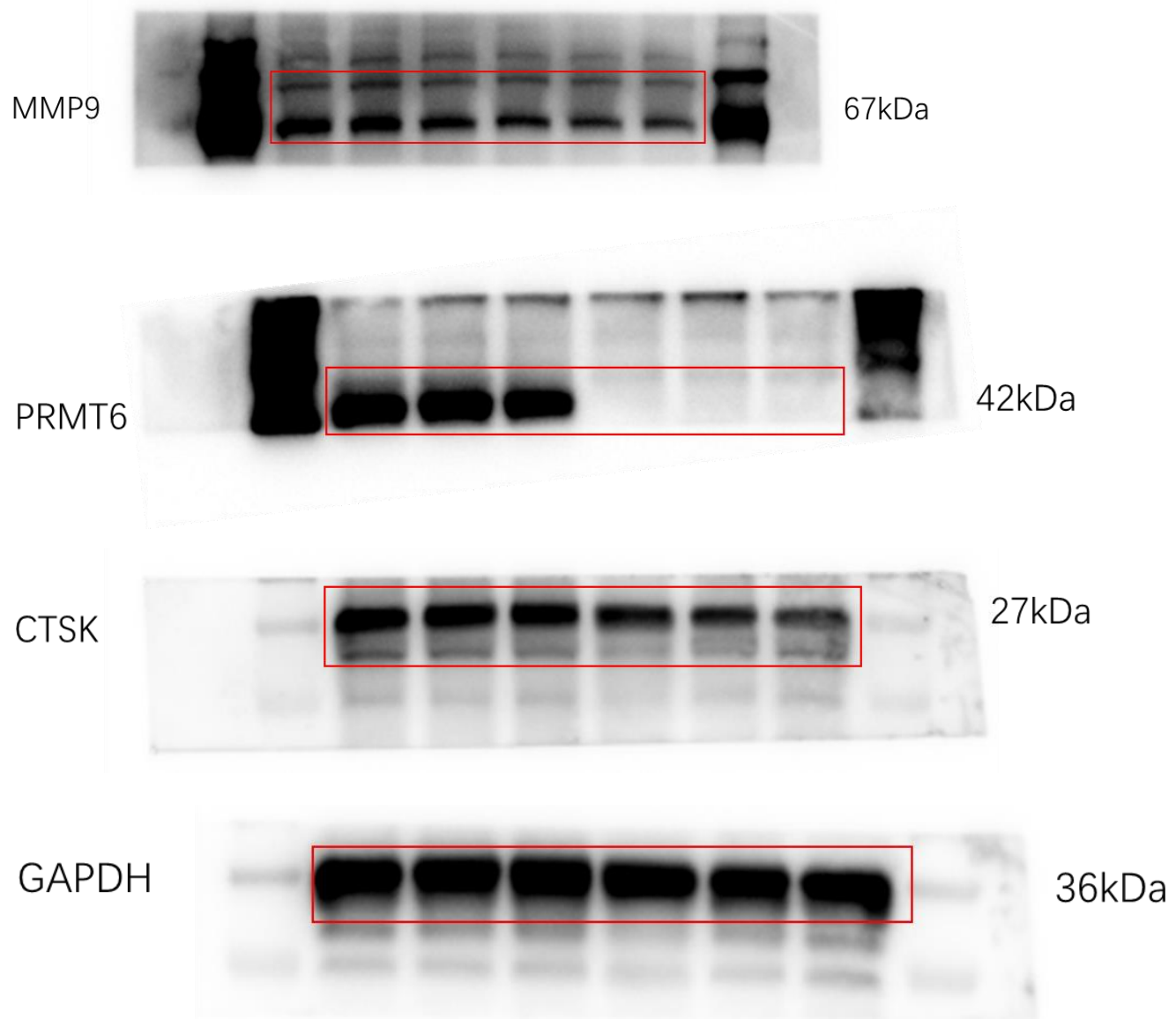

**Figure 5D**

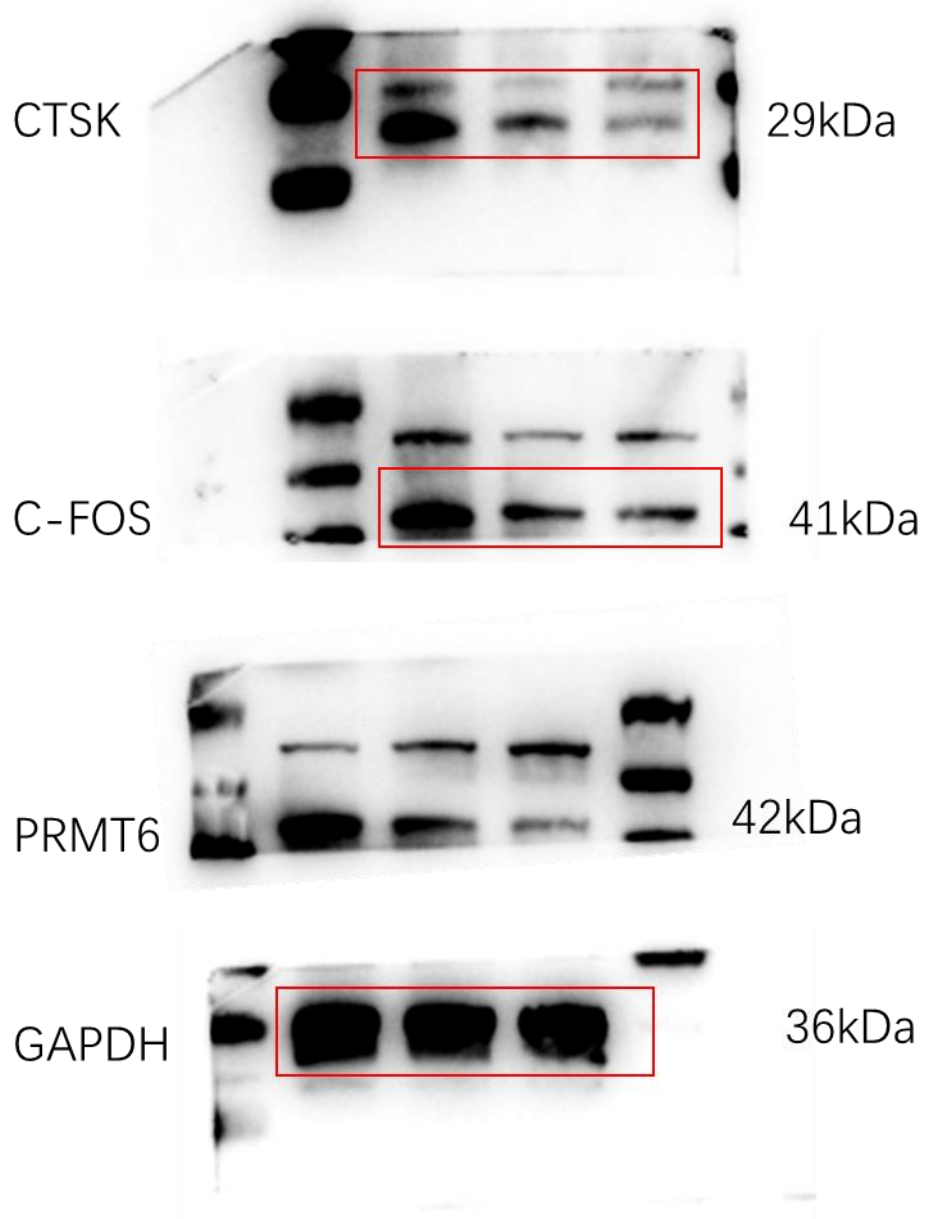

**Figure S1**

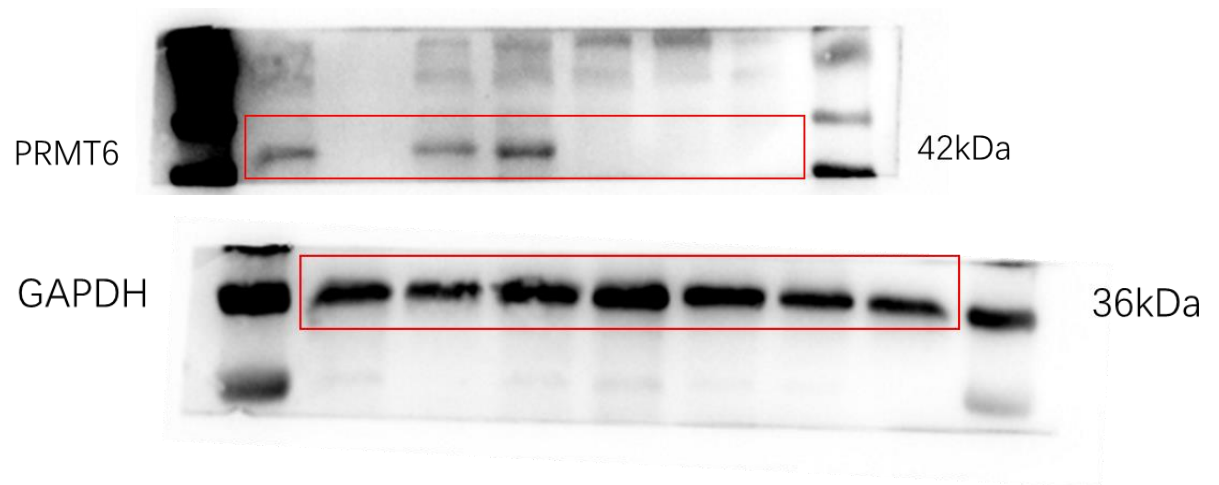

Supplement: Supplementary file 1 [file DataSheet1.pdf]
