# Supplementary material for: PRMT6 inhibitors promote fracture healing by modulating osteoclast glucose metabolism
Source: Front Immunol. 2025 Sep 18;16:1637232. doi: 10.3389/fimmu.2025.1637232 (PMC12488595; doi:10.3389/fimmu.2025.1637232)
Supplement: Supplementary file 2 [file DataSheet2.docx]

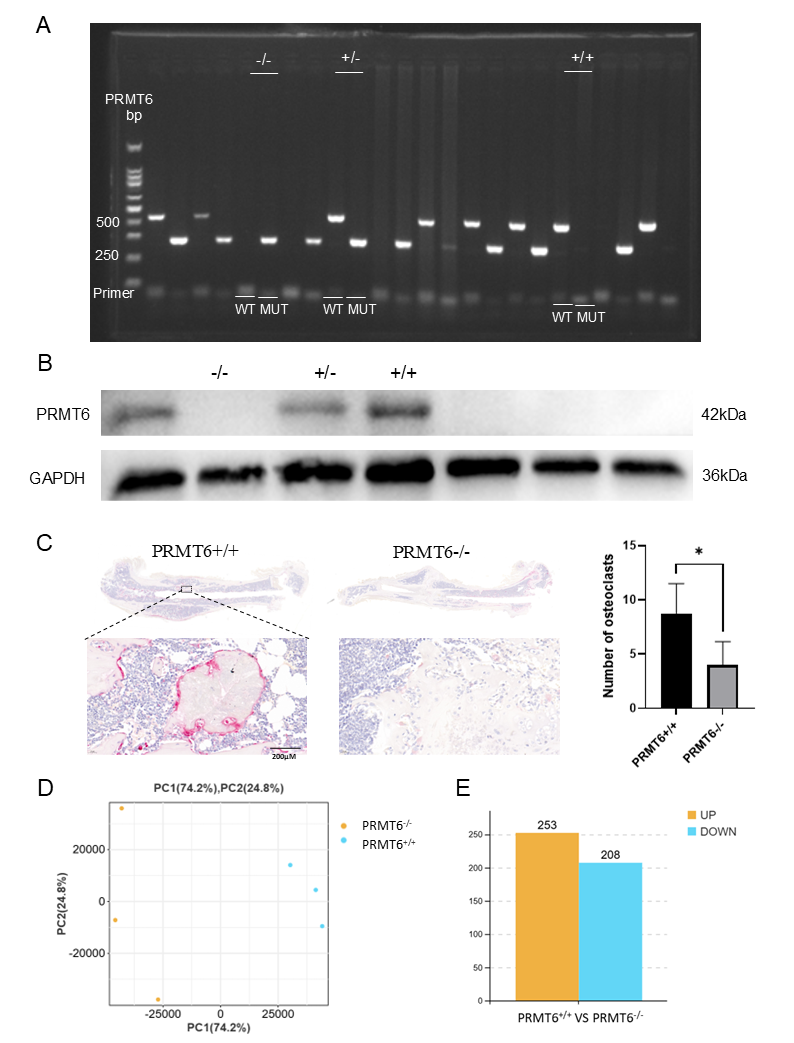


**Figure S1 (A)** DNA electrophoresis analysis of mouse gene genotype. **(B)** Western blot to verify the genotype of mouse genes. **(C)** Representative TRAP staining images of the PRMT6+/+group and the PRMT6-/-group, quantitative analysis of the number of osteoclast. **(D,E)** Principal component analysis (PCA) and Column chart of DEGs from RNA sequencing in +/+ mice and PRMT6-/- mice three weeks after fracture.


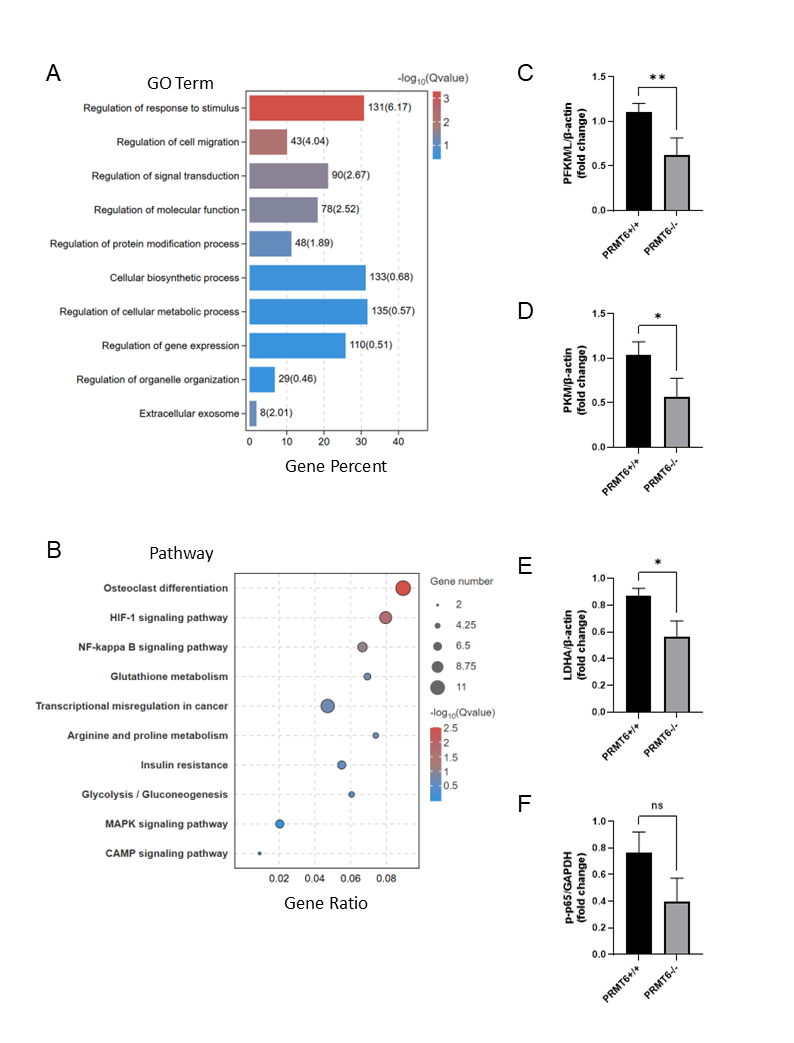


**Figure S2 (A,B)** Gene Ontology, GO enrichment analysis in RNA sequencing of PRMT6 +/+ mice and PRMT6-/- mice three weeks after fracture revealed significantly enriched biological processes and KEGG enrichment analysis of genes associated with differential peaks between the two groups. **(C-F)** Western blotting was conducted to detect the expression of PFKM/L,PKM,LDHA and p-p65 in RANKL-induced BMMs from PRMT6 +/+ group and PRMT6-/- group.


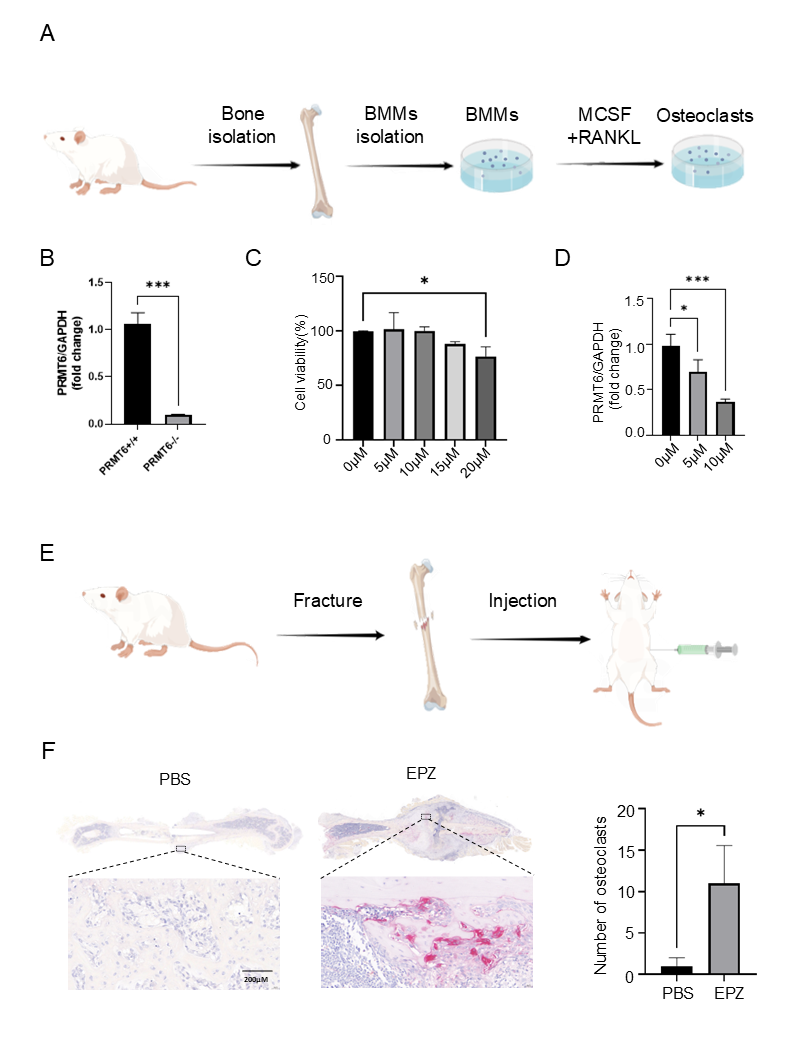


**Figure S3 (A)** Schematic diagram of osteoclast induction process. **(B)** Western Blotting demonstrates the absence of PRMT6. **(C)** BMMs were cultured in α‑MEM containing various concentrations of PRMT6 for 24h, and cell viability was assessed using Cell Counting Kit‑8 assays. **(D)** Western Blotting demonstrates that PRMT6 is inhibited to varying degrees **(E)** Schematic diagram of intraperitoneal injection administration process in C57 mice after fracture. **(F)** Representative TRAP staining images of the inhibitor group and the PBS group, quantitative analysis of the the number of osteoclast.
